# Acute Catatonia Following a Cerebellar Stroke: A Case Report

**DOI:** 10.7759/cureus.69645

**Published:** 2024-09-18

**Authors:** Shawna Koprucki, Roy Morcos

**Affiliations:** 1 Family Medicine, Mercy Health St. Elizabeth Boardman Hospital, Boardman, USA

**Keywords:** catatonia, cerebellar stroke, lorazepam challenge, lorazepam challenge test, motor rigidity, speech disturbance

## Abstract

Catatonia can present with a wide spectrum of psychomotor symptoms and should be considered in the differential diagnosis of hospitalized patients with speech and motor difficulties. Catatonia is defined as the presence of three or more of the following: catalepsy, waxy flexibility, stupor, agitation, mutism, negativism, posturing, mannerisms, stereotypies, grimacing, echolalia, and echopraxia. In this case, a 72-year-old black woman was admitted with difficulty with speech and ambulation and found to have a cerebellar stroke on a brain MRI. However, her symptoms of variable rigidity, mutism, and marked psychomotor slowing were not attributable to the small left-sided cerebellar infarction on imaging. A dramatic response to a lorazepam challenge confirmed a diagnosis of acute catatonia secondary to a medical condition.

## Introduction

One of the first steps in evaluating a patient who presents with new-onset speech and motor deficits is to assess for an acute cerebrovascular accident (CVA). However, discovering a CVA should not conclude the diagnostic evaluation. Patients with psychomotor symptoms such as speech changes, staring, or changes in tone and posture should be suspected to have acute catatonia [1]. It is defined as the presence of three or more of the following: catalepsy, waxy flexibility, stupor, agitation, mutism, negativism, posturing, mannerisms, stereotypies, grimacing, echolalia, and echopraxia [1]. Though catatonia was previously thought to be secondary to psychiatric illnesses, the DSM-5 also considers that it may be due to general medical conditions such as stroke [2]. It is critical to consider this diagnosis, as catatonia typically responds dramatically to pharmacotherapy and/or electroconvulsive therapy [3]. This case illustrates the importance of distinguishing between neurological conditions such as stroke, delirium, and catatonia. It also highlights that catatonia can be a sequela of stroke, particularly cerebellar stroke [4,5]. 

## Case presentation

**Figure 1:**
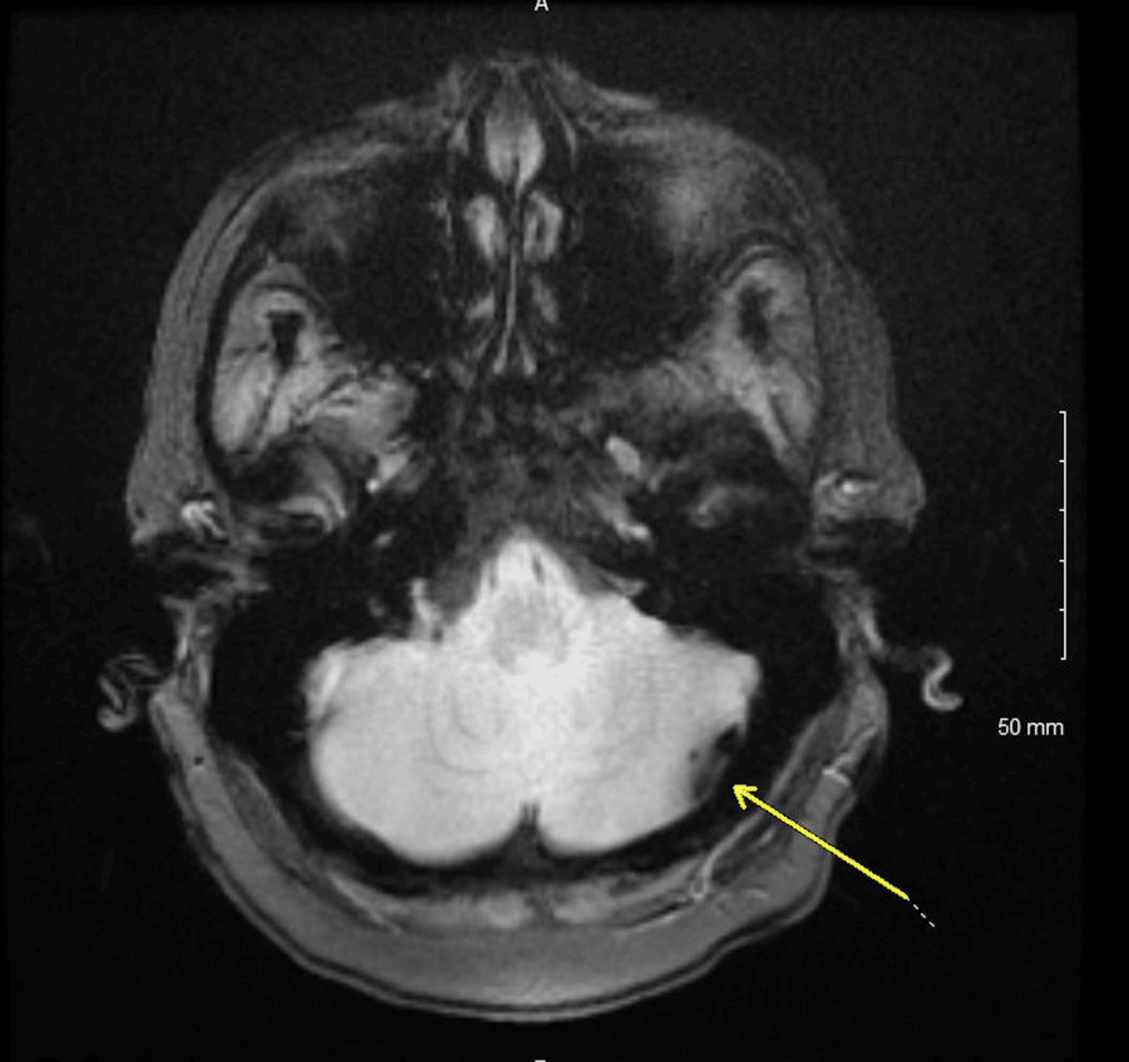
MRI brain without contrast (T2 weighted) with a yellow arrow indicating a small posterior left cerebellar stroke.

The patient is a 72-year-old female who presented with an inability to speak and ambulate for five days. A brain MRI without contrast revealed an acute small left-sided thrombotic cerebellar stroke (see Figure 1).

She was not a candidate for neurosurgical intervention due to her delayed presentation and was admitted to the medical unit. Her past medical history included type 2 diabetes, chronic kidney disease stage 4, hypertension, chronic depression, history of anal and vaginal cancer in remission, chronic hydronephrosis due to bladder obstruction, and osteoporosis. She had no history of alcohol, tobacco, or illicit drug use. Medications prior to admission included aspirin 81 mg, atenolol 25 mg daily, amlodipine 10 mg daily, atorvastatin 20 mg daily, glipizide 5 mg daily, magnesium oxide 400 mg daily, pantoprazole 40 mg daily and hydralazine 25 mg twice daily. Examination on admission revealed rigidity, manifested by resistance to passive movement of upper and lower extremities, weakness (grade 3-4/5) in the lower extremities, and mutism. Coordination could not be fully assessed due to weakness and muscle rigidity. Although she was unable to fully participate in the neurological examination, the small left-sided cerebellar stroke did not explain the findings. Her lack of speech is described as mutism, rather than a more specific motor or sensory aphasia, due to intermittent brief communication reported by ancillary staff, as well as lack of evidence of damage to language centers of the brain on neuroimaging. Laboratory testing showed evidence of acute kidney injury and hyperglycemia secondary to diabetes but no evidence of infection.

**Figure 2:**
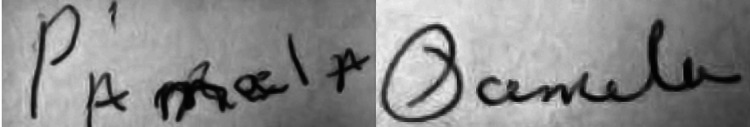
Handwriting sample immediately before (left) and after (right) lorazepam challenge. Used with permission.

The differential diagnosis included seizure disorder, delirium, and atypical catatonia. An MRA of the head revealed no significant stenosis or occlusion. An MRA of the neck was normal, and an EEG was consistent with a mild, nonspecific, waxing, and waning encephalopathy with no epileptiform discharges. An infectious process was ruled out. Her acute kidney injury improved with the administration of intravenous fluids. She continued to have variable psychomotor symptoms, including mutism, staring, negativism, rigidity consistent with Gegenhalten, and increased response latency in motor activity and speech. An example of this was noted during her speech and swallow evaluation, during which she was able to bring food to her mouth and swallow with sufficient dexterity, but this activity was so slow that she was unable to meet her caloric needs. A lorazepam challenge was performed at a dose of 1 mg I.V. with immediate improvement of her speech and motor symptoms. The rapid improvement in motor symptoms is illustrated by the handwriting sample obtained immediately before and after the lorazepam challenge (see Figure 2).

Her response to the lorazepam challenge confirmed a diagnosis of catatonia secondary to a medical condition, specifically the acute cerebellar stroke. 

Our patient’s response to lorazepam was dramatic, with an immediate return to baseline functioning. She was able to communicate clearly, and her movements were markedly more smooth and coordinated, with improvement in her ability to eat and drink. At the time of discharge, she required physical and occupational therapy for deconditioning. The lorazepam dose was adjusted from 1 mg three times daily to 0.5 mg three times daily due to sedation at the higher dose. At the outpatient follow-up visit approximately three months later, at which time she was still taking lorazepam, she was ambulatory, living independently, and back to her neurological baseline with no evidence of residual rigidity or weakness. 

## Discussion

Initially thought to be associated only with psychiatric disorders, catatonia is relatively common among elderly adults with a variety of medical conditions, with a prevalence estimated at around 9% or higher in older hospitalized adults [2,6,7]. It should be suspected in patients with psychomotor symptoms, including changes in speech, staring, or changes in tone and posture. Correctly identifying and treating this condition can positively impact patients' care since catatonia typically responds dramatically to pharmacotherapy and/or electroconvulsive therapy [3]. Although catatonia can coexist with delirium, it is essential to distinguish between the two conditions as the pharmacological treatments differ. The hallmarks of delirium include fluctuating mental status and level of consciousness, inattention, and disorganized thinking, which were not key features of our patient's symptomatology, though staring could be interpreted as a sign of inattention. Initial treatment of catatonia involves benzodiazepines, which can aggravate delirium, whereas delirium with aggressive behavior or agitation is sometimes treated with antipsychotics, which can aggravate catatonia [4]. 

In a patient presenting with a sudden speech impediment, a cerebrovascular accident involving the language centers of the cerebral cortex is always a major consideration. A brain MRI effectively ruled out this possibility in our patient. As a result, catatonia with mutism became a major consideration and was confirmed with a dramatic response to the lorazepam challenge.

Cases of catatonia following a cerebellar stroke have been previously reported [4,5]. In fact, it has been postulated that the cerebellum may be directly involved in the pathophysiology of catatonia due to its role in the medial motor loop and subsequent impaired control of self-initiated actions [5]. This suggests that the presence of a cerebellar stroke, in particular, should increase the index of suspicion for catatonia and heighten surveillance for associated psychomotor symptoms. 

Of the various psychomotor findings associated with catatonia, the most common include staring, stupor, mutism, and posturing, while the classic findings of catalepsy and waxy flexibility are noted less frequently [1]. In our patient, distinguishing aphasia from mutism, rigidity from weakness, and staring and stupor from delirium were the keys to arriving at the correct diagnosis. 

## Conclusions

In conclusion, catatonia is an important psychomotor syndrome that should not be considered a rare condition. Physicians need to be aware of catatonia both in the differential diagnosis of psychiatric conditions, delirium, and stroke, and as a sequela of stroke, particularly in a setting with limited availability of psychiatrists and neurologists. Appropriately diagnosing and treating catatonia can improve patient outcomes and quality of life, as seen in this case, in which a dramatic return of function and communication was witnessed after the appropriate intervention.
